# Biosynthesis of HLA-C heavy chains in melanoma cells with multiple defects in the expression of HLA-A, -B, -C molecules

**DOI:** 10.1038/sj.bjc.6690405

**Published:** 1999-05

**Authors:** A Martayan, R Fraioli, E Giorda, A Setini, G Ciccarelli, L Delfino, G B Ferrara, P Giacomini

**Affiliations:** Laboratory of Immunology, Regina Elena Institute CRS, Via delle Messi d'Oro 156, Rome, 00158, Italy; Laboratory of Immunogenetics, IST/CBA (Istituto Nazionale per la Ricerca sul Cancro/Centro di Biotecnologie Avanzate), Largo Benzi 10, Genova, 16132, Italy

**Keywords:** HLA-C, β_2_-microglobulin, calnexin, calreticulin, TAP, melanoma

## Abstract

Recent investigations have shown that malignant transformation may down-regulate the expression of class I HLA molecules, β_2_-microglobulin (β_2_m) and members of the antigen-processing machinery. In the present study, we HLA-genotyped and identified at a biochemical level the three (HLA-A25, -B8, -Cw7) class I alleles expressed by the previously described [D'Urso CM et al (1992) *J Clin Invest* **87**: 284–292] β_2_m-defective human melanoma FO-1 cell line and tested their ability to interact with calnexin, calreticulin and the TAP (transporter associated with antigen processing) complex. All these alleles were found to bind calnexin, but not calreticulin or the poorly expressed TAP complex, both in parental and β_2_m-transfected FO-1 cells, demonstrating a complex defect of class I expression in FO-1 cells. In these conditions, Cw7 heavy chains interacted with calnexin more strongly than A25 and B8, and preferentially accumulated in the endoplasmic reticulum, in both a calnexin-associated and a calnexin-free form. In addition, they could be transported to the cell surface at low levels even in the absence of β_2_m, without undergoing terminal glycosylation. These results establish a parallel between HLA-C and the murine D^b^ and L^d^ molecules which have been found to be surface expressed and functional in β_2_m-defective cells. They also demonstrate distinctive features of HLA-C molecules. We propose that the accumulation of several assembly intermediates of HLA-C might favour the binding of peptide antigens not readily bound by HLA-A and -B molecules in neoplastic cells with suboptimal class I expression. © 1999 Cancer Research Campaign


*British Journal of Cancer* (1999) **80**(5/6), 639–649
© 1999 Cancer Research Campaign
Article no. bjoc.1998.0405

### Biosynthesis of HLA-C heavy chains in melanoma cells with multiple defects in the expression of HLA-A, -B, -C molecules

**A Martayan[1], R Fraioli[1], E Giorda[1], A Setini[1], G Ciccarelli[1], L Delfino[2], GB Ferrara[2] and P Giacomini[1]**

[1]Laboratory of Immunology, Regina Elena Institute CRS, Via delle Messi d'Oro 156, 00158 Rome, Italy; [2]Laboratory of Immunogenetics, IST/CBA (Istituto Nazionale per la Ricerca sul Cancro/Centro di Biotecnologie Avanzate), Largo Benzi 10, 16132 Genova, Italy

**Summary** Recent investigations have shown that malignant transformation may down-regulate the expression of class I HLA molecules, $\beta_2$-microglobulin ($\beta_2$m) and members of the antigen-processing machinery. In the present study, we HLA-genotyped and identified at a biochemical level the three (HLA-A25, -B8, -Cw7) class I alleles expressed by the previously described [D'Urso CM et al (1992) *J Clin Invest* **87**: 284–292] $\beta_2$m-defective human melanoma FO-1 cell line and tested their ability to interact with calnexin, calreticulin and the TAP (transporter associated with antigen processing) complex. All these alleles were found to bind calnexin, but not calreticulin or the poorly expressed TAP complex, both in parental and $\beta_2$m-transfected FO-1 cells, demonstrating a complex defect of class I expression in FO-1 cells. In these conditions, Cw7 heavy chains interacted with calnexin more strongly than A25 and B8, and preferentially accumulated in the endoplasmic reticulum, in both a calnexin-associated and a calnexin-free form. In addition, they could be transported to the cell surface at low levels even in the absence of $\beta_2$m, without undergoing terminal glycosylation. These results establish a parallel between HLA-C and the murine $D^b$ and $L^d$ molecules which have been found to be surface expressed and functional in $\beta_2$m-defective cells. They also demonstrate distinctive features of HLA-C molecules. We propose that the accumulation of several assembly intermediates of HLA-C might favour the binding of peptide antigens not readily bound by HLA-A and -B molecules in neoplastic cells with suboptimal class I expression.

**Keywords:** HLA-C; $\beta_2$-microglobulin; calnexin; calreticulin; TAP; melanoma

Class I MHC molecules (including H2-D, -K, -L in the mouse and HLA-A, -B, -C in humans) are comprised of a heavy (44 kDa) chain (which is glycosylated and highly polymorphic) and a light (12 kDa) non-polymorphic chain termed $\beta_2$-microglobulin ($\beta_2$m). These two subunits assemble in the endoplasmic reticulum (ER) in the presence of short antigenic peptides (mostly 9-mers) and with the aid of molecular chaperones and cellular proteins including calnexin, calreticulin and the TAP1:TAP2 (transporter associated with antigen processing) complex. Distinct class I assemblies interact with these cellular proteins through stages of biosynthesis (Hansen and Lee, 1997). In humans, calnexin preferentially interacts with $\beta_2$m-free, unfolded heavy chains. Calreticulin and the TAP complex bind to $\beta_2$m-associated heavy chains and facilitate the folding and peptide loading of the class I complex, which is then transported to the cell surface for recognition by cytotoxic T-lymphocytes (CTLs) and natural killer (NK) cells. During transport, the core glycosylated heavy chain undergoes maturation and sialyc acid moieties are added at last in the Golgi compartment (Hansen and Lee, 1997).

The $\beta_2$m subunit has been shown to be essential for the correct assembly, biosynthesis and transport to the cell surface of most class I molecules (Fellous et al, 1977; Hansen and Lee, 1997). More recently, however, using antibodies capable of discriminating different conformations of class I molecules, several murine class I heavy chains, including $D^b$, $L^d$ and $K^b$ molecules, have been detected at low levels on the surface of normal as well as transformed cells lacking the $\beta_2$m subunit and/or the TAP complex (Potter et al, 1984; Allen et al, 1986; Smith et al, 1993; Machold et al, 1995). These heavy chains, 'free' of $\beta_2$m, have been demonstrated to be either 'unfolded' (Allen et al, 1986; Smith et al, 1993), or at least partially folded (Machold et al, 1995), and to bear mature carbohydrates (Hansen et al, 1988; Machold et al, 1995). Biochemical and functional data in $\beta_2$m knockout mice are consistent with the idea that both $D^b$- and $L^d$-free heavy chains may retain some ability to bind (Smith et al, 1993) and present (Lehmann-Grube et al, 1994; Cook et al, 1995) antigen even when synthesized in the absence of $\beta_2$m. In contrast, there is no evidence that any of the more than 300 sequenced HLA-A and -B alleles might be constitutively expressed on the cell surface of most (Fellous et al, 1977; D'Urso et al, 1992; Gattoni-Celli et al, 1992; Wang et al, 1993; Bicknell et al, 1994; Porgador et al, 1997) $\beta_2$m-defective cells of human origin.

It has been long known that heavy chains unable to bind $\beta_2$m and/or peptide (Elliott, 1991) are retained in early exocytic compartments to be subsequently degraded (Degen and Williams, 1991; Rajagopalan and Brenner, 1994). However, the efficiency of the retention and degradation mechanisms has been found to be different in different class I molecules. For instance, murine H2-$D^b$ (Allen et al, 1986), -$L^d$ (Smith et al, 1993) and human HLA-C (Neefjes and Ploegh, 1988) heavy chains display a low binding to

*Received 14 April 1997*
*Revised 4 November 1998*
*Accepted 6 November 1998*

*Correspondence to:* P Giacomini

$\beta_2$m and have the longest reported half-lives as free heavy chains.

It has been proposed that clones of neoplastic cells unable to express one or more class I alleles might arise and be selected in vivo, in the immunocompetent host, possibly because they are provided with a distinctive ability to evade the immune surveillance (Garrido et al, 1993). Thus, the biochemical characterization of the various class I molecules in class I-defective cells bears implications on the mechanisms by which the various immune effectors control neoplastic cell growth.

In the present report, we have studied the conformation, intracellular transport and expression of class I molecules in the $\beta_2$m-defective FO-1 melanoma cell line (D'Urso et al, 1992) and its $\beta_2$m transfectants. To this end, we have used monoclonal antibodies detecting free and $\beta_2$m-associated HLA-A, -B, -C heavy chains and have taken advantage of a recently characterized (Setini et al, 1996) monoclonal antibody, named L31. This antibody detects a distinctive conformation of the α1 domain α helix surrounding the peptide binding groove of most HLA-C, and a few HLA-B, alleles when free of $\beta_2$m (Setini et al, 1996). We provide evidence herein for a complex multifactorial defect impairing class I expression in FO-1 cells, for a distinctive biosynthesis of HLA-C molecules and for their ability to be expressed on the surface of FO-1 cells even in the absence of $\beta_2$m.

### MATERIALS AND METHODS

### Cell lines and nucleic acid biochemistry

The $\beta_2$m-defective FO-1 melanoma cell line and its serological HLA typing (A25, -B8) have been previously described (D'Urso et al, 1992). The HLA-A, -B, -C genotype of FO-1 was assessed by locus-specific polymerase chain reaction (PCR) amplification of genomic DNA followed by second and third exon direct sequencing, as described (Pera et al, 1997). The isoelectrofocussing (IEF) pattern (Giacomini et al, 1997) and the HLA-A, -B, -C genotype (Prasad and Yang, 1996) of the HLA homozygous MGAR B-cell line have been published. The HLA-A, -B, -C negative 721.221 cell line (221 hereafter) (Shimizu and DeMars, 1989) and its Cw*0102 transfectants (Setini et al, 1996), the TAP1/TAP2-defective cell line 174 × CEM.T2 (T2 hereafter) and its parental counterpart T1 (Salter and Cresswell, 1986) have been described. To increase the expression of TAP molecules, FO-1 cells were treated with recombinant interferon (IFN)-γ at the optimal concentration of 100 U $ml^{-1}$ for 72 h.

A full length cDNA for human $\beta_2$m was obtained as described (Martayan et al, 1997). The pCMV-$\beta_2$m plasmid and the empty pCMV–neo vectors (20 µg) were linearized with Xmn I and transfected by lyposome-mediated DNA transfer (Boehringer Mannheim, Germany). Stable transfectants were isolated on the basis of their ability to grow in 750 µg $ml^{-1}$ G418 (Gibco BRL, Gaithersburg, MD, USA). Individual colonies were recovered in cloning cylinders and expanded.

### Immunochemical methods

The following mouse monoclonal antibodies to class I molecules were used: Namb-1 is to $\beta_2$m (Pellegrino et al, 1982); W6/32 is to conformed HLA class I molecules associated with $\beta_2$m (Parham et al, 1979); L31 identifies an epitope present on all the serologically defined HLA-C (Cw1 through Cw7) and on certain HLA-B heavy chains (including B8) after denaturation. Pulse-chase studies have shown that the L31 epitope becomes 'hidden' upon assembly with $\beta_2$m, this process being more efficient in HLA-B than HLA-C heavy chains. HLA-A heavy chains are essentially unreactive with L31 (Setini et al, 1996; Giacomini et al, 1997). HC-10 was raised against denatured HLA-B locus heavy chains and cross-reacts with certain HLA-C and -A products including B8 and A25 (Stam et al, 1986). A monoclonal antibody to 72-kDa heat shock protein (Amersham, Little Chalfont, UK) and rabbit polyclonal antibodies to the C-terminal sequences of calnexin and calreticulin (Stressgen, Victoria, Canada) were also used. The polyclonal antibodies to TAP1 and TAP2 were generous gifts of Dr J Trowsdale. Flow cytometry, metabolic labelling and one-dimensional IEF have been previously described (Yang, 1989; Setini et al, 1996). Western blotting experiments were performed as described (Setini et al, 1996) using chemiluminescence as a detection method. Cell surface iodination was carried out by Iodogen beads (Pierce, Rockford, IL, USA) in conditions reported (Markwell and Fox, 1978) to limit the access of $^{125}$I to surface structures. In certain control experiments, iodination was performed on cell extracts. Cells were lysed by hypotonic shock in 10 mM sodium chloride, 5 mM magnesium chloride, 1 mM dithiothreitol and 10 mM Tris pH 7.4, and disrupted by 20 strokes of a Dounce homogenizer. The postnuclear supernatants were radiolabelled in parallel with whole viable cells using the Iodogen beads, then adjusted to 1% NP40 (final concentration) and immunoprecipitated.

### RESULTS

### HLA-A, -B, -C genotyping of FO-1 cells

HLA-A and -B DNA fragments were PCR amplified from genomic DNA of FO-1 cells and sequenced. The previously defined HLA-A25 and -B8 specificities (D'Urso et al, 1992) were found to correspond to the HLA-A*2501 and -B*0801 alleles. HLA-C, for which no tissue typing is available, was genotyped as HLA-Cw*0701. Hereafter, these alleles will be referred to as HLA-A25, B8 and -Cw7. No other HLA-A, -B or -C specific DNA fragments could be amplified from FO-1 cells.

### Allele assignment of HLA-A, -B, -C heavy chain components synthesized by FO-1 cells

To identify the three genotyped class I alleles at the protein level and assess their biochemical features both in the presence and absence of $\beta_2$m, FO-1 cells were transfected with either the pCMV-$\beta_2$m or the empty pCMV–neo vector. Two G418-resistant colonies of FO-1 cells from each transfection experiment were metabolically labelled and immunoprecipitated with antibodies to $\beta_2$m-free and $\beta_2$m-associated heavy chains. These preliminary experiments identified two different patterns of antibody reactivity, exclusively dependent on the presence or absence of $\beta_2$m (not shown). Because representative of the two patterns of antibody reactivity, parental (FO-1–PT) cells and one $\beta_2m^+$ transfectant (FO-1–$\beta_2$m) were selected to be employed in further biochemical studies.

To distinguish the different class I heavy chains after immunoprecipitation, we took advantage of one-dimensional IEF, a conventional method to resolve the different HLA-A, -B, -C alleles (Neefjes et al, 1986). FO-1 parental cells, their $\beta_2$m

 © Cancer Research Campaign 1999

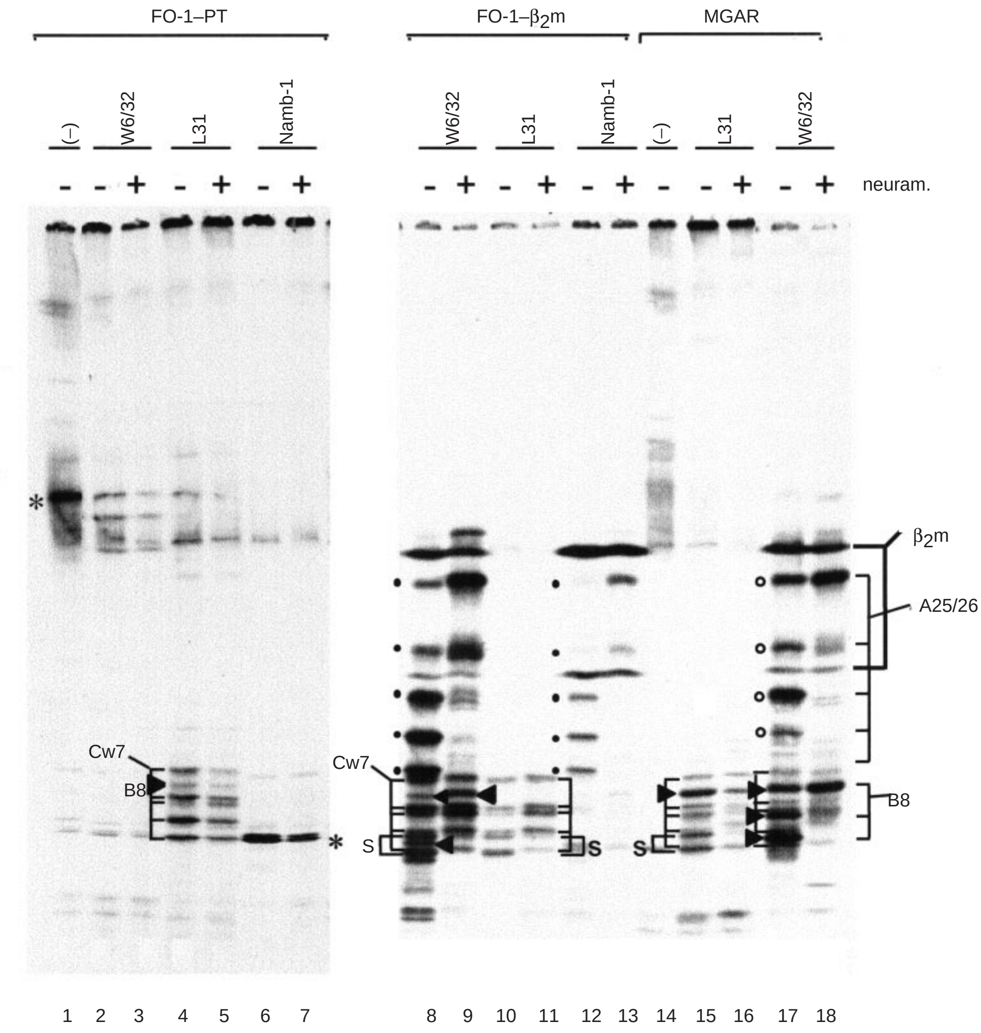

**Figure 1** Immunochemical analysis of class I HLA molecules synthesized by FO-1 cells and their $\beta_2$m transfectants. Parental FO-1 cells (FO1-PT), one clone transfected with the pCMV–$\beta_2$m plasmid (FO-1–$\beta_2$m) and the MGAR B-lymphoid cell line, as indicated, were metabolically labelled for 2 h with $^{35}$S-methionine. Soluble extracts prepared with the non-ionic detergent NP40 were immunoprecipitated with immunoadsorbents covalently linked to the following antibodies: W6/32 to $\beta_2$m-associated heavy chains, L31 to $\beta_2$m-free heavy chains and Namb-1 to $\beta_2$m, as indicated. Immunoadsorbents conjugated with immunoglobulins of irrelevant specificity (−) were used as negative controls. Immunoprecipitates were analysed by IEF (bottom: acidic end) both undigested (−) and digested (+) with neuraminidase. Neuraminidase-insensitive and -sensitive (s = sialylated) HLA-Cw7 forms are indicated. Asterisks (*) identify background bands, present in the control immunoprecipitates. These bands are prominent in the absence of specific immunoprecipitates. Dots (●), open circles (○), and arrowheads (▶) indicate the HLA-A25, HLA-A26 and HLA-B8 components respectively. HLA-A25 heavy chains in FO-1–$\beta_2$m cells display one acidic band which is not present in the A26 heavy chains expressed by MGAR cells (see for instance lanes 12 and 17)

transfectants and the partially HLA-matched (Prasad and Yang, 1996) MGAR cell line (HLA-A*2601, -B*0801, Cw*0701; from here on referred to as A26, B8 and Cw7) whose IEF pattern we recently characterized (Giacomini et al, 1997) were metabolically labelled for 2 h with $^{35}$S-methionine, solubilized by the non-ionic detergent NP40 and immunoprecipitated with antibodies Namb-1 to $\beta_2$m, W6/32 to $\beta_2$m-associated heavy chains and L31 to $\beta_2$m-free heavy chains. In order to reduce the charge heterogeneity of class I heavy chains and characterize these molecules in their sialylation (i.e. in their intracellular transport), class I immunoprecipitates were analysed by IEF both undigested and digested with neuraminidase.

From the results presented in Figure 1, lanes 8–18, it may be noted that MGAR and FO-1–$\beta_2$m cells gave rise to remarkably similar IEF patterns with all the antibodies used. This was expected, since the two cell lines are HLA-B/-C identical and the A25 and A26 alleles are known (Yang, 1989) to comigrate in IEF. The B*0801 allele expressed by both FO-1–$\beta_2$m and MGAR cells had the limited pI heterogeneity of HLA-A and -B alleles and migrated as 2 and 3 regularly spaced bands respectively (Figure 1, lanes 8 and 17: arrowheads). Upon neuraminidase digestion only the unsialylated, most basic band remained in both cells (Figure 1, lanes 9 and 18), identifying the HLA-B*0801 polypeptides in the two cell lines and demonstrating their identity. The remaining bands in the low (acidic) region of the gel were assigned to HLA-Cw*0701. In contrast to HLA-A and -B alleles (Hajek-Rosenmayr et al, 1989; Setini et al, 1996), HLA-Cw7 displayed a multiplicity of irregularly spaced bands, five of which (marked as Cw7 in

© Cancer Research Campaign 1999

Figure 1) were (a) reactive with both W6/32 and L31, (b) insensitive to neuraminidase and (c) present in parental FO-1 (Figure 1, lanes 4–5) and FO-1–$\beta_2$m (Figure 1, lanes 8–11) cells. With the possible exception of the fourth band from the top, they were also present in MGAR cells (Figure 1, lanes 15–16). Their preferential reactivity with the HLA-C restricted L31 antibody, especially in FO-1 cells (Figure 1, lanes 4–5 and 10–11), supports their assignment to HLA-Cw7. It should be noted that some of these bands (i.e. the second and third from the top) closely migrate and are not fully resolved in certain IEF gels (see below). FO-1–$\beta_2$m and MGAR cells displayed all or most of the five Cw*0701 bands seen in the parental FO-1 cells as well as two acidic and neuraminidase-sensitive bands (marked as ‘s’) which were not seen in parental FO-1 cells (compare lanes 8–11 and 15–16 to lanes 4–5).

### Biochemical features of HLA-A, -B, -C molecules synthesized by FO-1 cells

Immunoprecipitation with Namb-1 (and other antibodies to $\beta_2$m, not shown) demonstrated the presence of $\beta_2$m in $\beta_2$m transfectants (Figure 1, lanes 12–13) and confirmed its absence in parental cells (Figure 1, lanes 6–7). Similarly, the W6/32 antibody, known to react with class I heavy chains only when complexed with $\beta_2$m (Parham et al, 1979), gave specific immunoprecipitates in the $\beta_2$m transfectants (Figure 1, lanes 8–9) but not in the $\beta_2$m-negative cells (Figure 1, lanes 2–3).

In addition to the classical uppermost $\beta_2$m component, one additional $\beta_2$m band could be seen in Namb-1 and W6/32 immunoprecipitates from all the $\beta_2$m-positive cells (Figure 1, lanes 8–9, 12–13, 17–18). Because this additional component is present in MGAR cells (Figure 1) and in wild-type cell lines of B-lymphoid or neoplastic origin (Setini et al, 1996; Giacomini et al, 1997), it does not represent an artifact resulting from plasmid construction or transfection. The nature of this component was not further investigated.

The levels of the $\beta_2$m components greatly exceeded those of the heavy chain when directly immunoprecipitated by the Namb-1 antibody to $\beta_2$m (Figure 1, lanes 12–13), but not when co-precipitated by the W6/32 antibody which recognizes heavy chains and $\beta_2$m in a defined (presumably 1:1) stoichiometric relationship (Figure 1, lanes 8–9), suggesting the presence of a large excess of free $\beta_2$m in $\beta_2$m transfectants. This excess was estimated to be ten to 20-fold by densitometry. Noticeably, HLA-B8 heavy chains were much less reactive with L31 in FO-1–$\beta_2$m (Figure 1, lanes 4, 5, 10, 11) than MGAR (Figure 1, lanes 15–16) cells.

From the above data, we may conclude that: (a) transfection of the $\beta_2$m cDNA rescued the expression of W6/32-reacting class I molecules in FO-1 cells; (b) HLA genotyping and protein biochemistry concordantly identified the HLA-A25, -B8 and -Cw7 alleles in FO-1 cells and each of the major class I heavy chain components could be assigned to an individual class I allele; (c) HLA-A, -B, -C alleles were sialylated only in the presence of $\beta_2$m; and (d) large amounts of HLA-Cw7 heavy chains were unable to assemble with $\beta_2$m. Presumably because not readily degraded, they accumulated in a free heavy chain form in FO-1 as well as in FO-1–$\beta_2$m cells. This occurred even in the presence of a large stoichiometric excess of $\beta_2$m. In contrast, HLA-B8 did not significantly accumulate in a free heavy chain form, suggesting that it was more efficiently assembled and/or disposed than Cw7. A practical consequence was that L31 behaved as an operationally HLA-C-specific antibody in metabolically labelled FO-1 cells.

### Steady-state levels of class I HLA molecules in FO-1 melanoma cells and their $\beta_2$m transfectants

Next we wanted to determine whether the accumulation of free HLA-Cw7 heavy chains could be explained by a preferential ability of these molecules to interact with calnexin, calreticulin and the TAP complex, thus accounting for their stability.

As a preliminary step to the assessment of protein:protein interactions, the steady-state levels of class I heavy chains, calnexin, calreticulin, TAP1 and TAP2 molecules were measured in total cell extracts of parental FO-1 and FO-1–$\beta_2$m cells. Soluble NP40 extracts were prepared, denatured in the presence of sodium dodecyl sulphate (SDS), boiled, resolved by SDS-polyacrylamide gel electrophoresis (SDS-PAGE), transferred to nitrocellulose filters and blotted with specific antibodies, as described in the legend to Figure 2. The HLA-A, -B, -C negative/$\beta_2$m-positive 221 B cell line, FO-1 cells treated with IFN-γ, the TAP1/TAP2-negative T2 cell line, and its parental counterpart T1, were run as controls. As shown in Figure 2A, the steady-state levels of the L31-reactive class I heavy chains in FO-1 cells (L31 binds both Cw7 and B8 after heavy chain denaturation, see Materials and Methods) were slightly greater in the presence than in the absence of $\beta_2$m (possibly a result of the greater stability of heavy chain: $\beta_2$m complexes as compared to free heavy chains, see below), and in the presence than in the absence of IFN-γ (Figure 2, lanes 1–4). However, they remained invariably much lower than in the HLA-A, -B, -C expressing lymphoid cell lines (Figure 2, lanes 6–7). Interestingly, while calnexin (Figure 2, lanes 15–21) and calreticulin (Figure 2, lanes 22–28) were expressed at similar levels in all the cell lines, the TAP1 (Figure 2, lanes 8–14) and TAP2 (not shown) subunits were expressed at much lower levels in melanoma than in lymphoid cells. In Figure 2, they were detectable after treatment with IFN-γ or, in untreated cells, after long exposures of the filter (Figure 2, lanes 8 and 10). In agreement with the results presented in Figure 1, $\beta_2$m was not a limiting factor in class I assembly since it was present in comparable amounts in all the $\beta_2$m-positive cells (Figure 2, lanes 31–35). The FO-1 melanoma cells and the control lymphoid cell lines therefore express the two promiscuous chaperones calnexin and calreticulin at similar high levels, while FO-1 cells selectively lack the class I-dedicated chaperone (Solheim et al, 1997a), TAP.

### Interactions of class I heavy chains with proteins of the antigen processing machinery

It has been previously shown that calnexin binds $\beta_2$m-free class I heavy chains in parental FO-1 cells (Rajagopalan and Brenner, 1994). However, the effect of transfection with $\beta_2$m on this interaction has not been assessed. Also unknown are the relative abilities of the different heavy chain alleles to bind calnexin, calreticulin and, in FO-1 cells, TAP. To address these issues, soluble cell extracts were prepared in the mild non-ionic detergent CHAPS, known to preserve protein:protein interactions. We found CHAPS to be superior to digitonin in preliminary experiments. CHAPS extracts from FO-1 and FO-1–$\beta_2$m cells were immunoprecipitated with antibodies to TAP1, calnexin and calreticulin. The resulting immunoprecipitates were run on a SDS-PAGE gel and Western blotted. The filters were stained with antibodies to class I heavy chains and with control antibodies. Parental 221 cells and the 221.Cw*0102 transfectant were included as controls. It was readily evident from this experiment (Figure 2 B–D) that class I

 © Cancer Research Campaign 1999

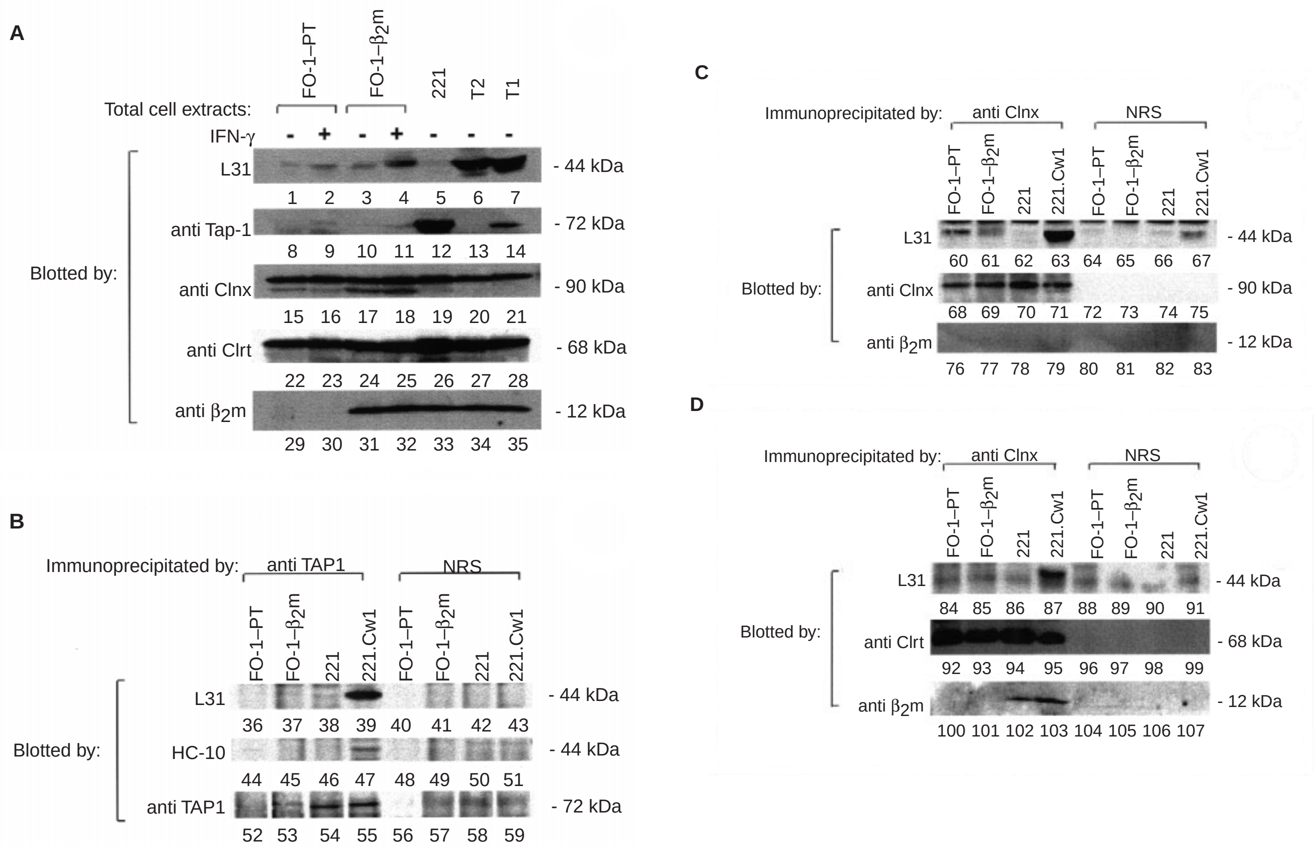

**Figure 2** HLA-class I molecules in FO-1 cells: steady-state levels and interactions with members of the antigen-processing machinery. Soluble extracts of the FO-1-PT and FO-1–$\beta_2$m melanoma cells, and of the 221, T2 and T1 lymphoid cell lines were prepared with the non-ionic detergent CHAPS. (**A**) A total of 100 µg of cell extract per lane were submitted to SDS-PAGE (12.5% acrylamide, reducing conditions) and blotted onto a nitrocellulose filter. The filter was stained/stripped five consecutive times with the indicated antibodies. The relevant areas of the filter displaying specific signals are shown. In the remaining panels, the same extracts were immunoprecipitated by polyclonal rabbit antibodies to TAP1 (**B**), calnexin (**C**) and calreticulin (**D**), and, as a control, by normal rabbit serum (NRS, **B–D**). Immunoprecipitates were resolved by SDS-PAGE (12.5%) and Western blotted. The resulting filters were stained with the indicated antibodies. Bands of the expected sizes were identified by the various antibodies in selected areas of the filters heavy chains interacted with calnexin not only in $\beta_2$m-defective FO-1 cells but also, although to a lesser extent, in FO-1–$\beta_2$m cells (Figure 2C, lanes 60–61). In both cases these interactions involved the $\beta_2$m-free heavy chains, as assessed by the absence of staining with an antibody to $\beta_2$m in the same filter (Figure 2, lanes 76–77).

In sharp contrast, essentially no HLA-A25, B8 and Cw7 heavy chains were detectable, using two distinct antibodies to class I molecules (see Materials and Methods for antibody specificities), in association with either TAP1 (Figure 2, lanes 36–37, 44–45) or calreticulin (Figure 2, lanes 84–85 and data not shown). This was not unexpected in the case of the TAP complex, which was expressed at low and practically undetectable steady-state levels (see Figure 2, lanes 52 and 53 as compared to lanes 54 and 55; also see lanes 8 and 10), but was quite surprising in the case of the high abundance protein calreticulin (Figure 2, lanes 92–93). The procedure we used to detect protein:protein interactions was sufficiently sensitive, since calreticulin was found to associate with HLA-Cw1 heavy chains in 221.Cw1 cells (Figure 2, lane 87) and with $\beta_2$m, as described (Solheim et al, 1997*b*), in the HLA-A, -B, -C negative 221 cells (Figure 2, lane 102). Thus, the poor association of calreticulin with class I heavy chains was not due to technical reasons. Co-immunoprecipitation experiments from metabolically labelled cell extracts (not shown) confirmed that A25, B8 and Cw7 heavy chains are unable to bind calreticulin.

From these data we may conclude that, even after reconstitution with $\beta_2$m, class I molecules are synthesized in FO-1 cells in the presence of limiting amounts of the TAP complex. In addition, they fail to efficiently interact with the high abundance calreticulin protein. This indicates that the defect in class I expression of FO-1 cells is more complex than expected. In line with this interpretation, flow cytometry reproducibly (three separate experiments) demonstrated that the W6/32-reactive molecules present at the cell surface of FO-1–$\beta_2$m cells cultured at 26°C for 18 h were from 25% to 50% more abundant than those in cells cultured at 37°C (not shown). Stabilization by hypothermia is a common feature in cells unable to properly assemble class I complexes (Elliott, 1997).

Because the only detectable interaction of class I molecules in FO-1 cells is with calnexin, we sought to determine to what extent the three class I alleles expressed in FO-1 cells interact with this molecular chaperone and to assess the time course of these interactions.

### Pulse-chase analysis of FO-1 melanoma cells

The calnexin:heavy chain interaction was studied by a 5-min pulse with $^{35}$S-methionine followed by variable periods of chase, up to 180 min (Figure 3). CHAPS lysates were immunoprecipitated

*© Cancer Research Campaign 1999* 

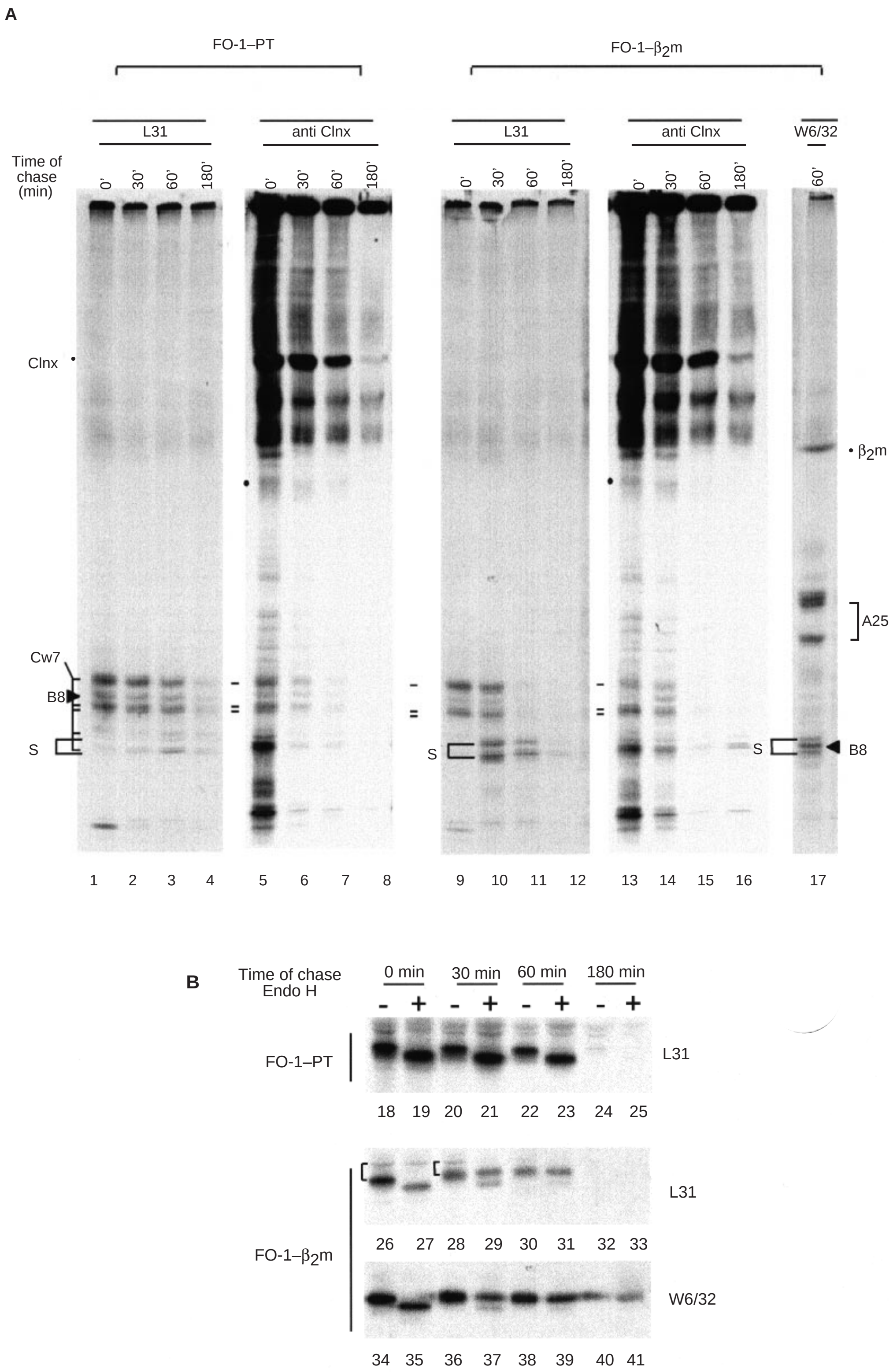

**Figure 3** Pulse chase analysis of FO-1-PT and FO-1–$\beta_2$m cells. The FO-1–PT and FO-1–$\beta_2$m melanoma cells were pulsed for 5 min with $^{35}$S-methionine (approximately 17.5 MBq ml$^{-1}$), then chased for the indicated times and lysed by the non-ionic detergent CHAPS. In (**A**) the soluble extracts were immunoprecipitated with the L31 murine monoclonal antibody and a polyclonal antibody to calnexin. The resulting immunoprecipitates were resolved by IEF. A control immunoprecipitate with the W6/32 antibody is also shown (for further details see the legend to Figure 1); in (**B**) the immunoprecipitates were prepared with the L31 and W6/32 monoclonal antibodies and resolved by SDS-PAGE (12.5% acrylamide, reducing conditions). To monitor the intracellular transport of class I molecules, immunoprecipitates were prepared in duplicate and either treated (+) by endoglycosidase H (Endo H) which cleaves immature glycans or mock-treated (–). Bars in lanes 26 and 28 measure the distance of the class I heavy chains from a background band present in most lanes. The autoradiography of the gel shown in lanes 18–25 lasted longer (72 h) than those of the gels depicted in the remaining lanes (24 h)

 *© Cancer Research Campaign 1999*

with L31 and with an antibody to calnexin, then analysed by IEF (Figure 3A) and/or SDS-PAGE (Figure 3B) gels.

From the results shown in Figure 3, it appears that calnexin migrated to a basic pl, as experimentally determined for this protein (see information on the Internet at http://expasy.hcuge.ch/) and associated with a variety of protein bands in both parental (Figure 3, lanes 5–8) and $\beta_2$m-transfected (Figure 3, lanes 13–16) FO-1 cells. The bands comigrating with Cw1 were much more abundant than those corresponding to the A25 and B8 heavy chains (Figure 3, lanes 5 and 13). In agreement with the data in Figure 1, the HLA-Cw1 heavy chains were sialylated and acquired endoglycosidase H (Endo H) resistance only in the presence of $\beta_2$m (Figure 3, compare lanes 9–12 to lanes 1–4, and lanes 18–25 to lanes 26–41). As expected, sialylation and acquisition of Endo H resistance were simultaneous and coincided with a slight increase in the apparent molecular weight of the Cw7 heavy chains, clearly seen by comparing lane 26 to lane 28. These events mark the export of class I molecules from the ER.

In $\beta_2$m-expressing FO-1 melanoma cells, export from the ER correlated, as expected, with the release of Cw7 heavy chains from calnexin (Figure 3, lanes 9–16). In contrast, large amounts of L31-reacting Cw7 heavy chains were present in the ER of $\beta_2$m-defective cells and remained at similar high levels throughout the first 60 min of chase (Figure 3, lanes 1–3), well after the nearly complete disappearance of the calnexin:heavy chain complexes (Figure 3, lanes 5–7). Remarkably, $\beta_2$m-free Cw7 heavy chains in parental and $\beta_2$m-transfected FO-1 cells, although bona fide located in different subcellular compartments, were equally stable (Figure 3, lanes 18–33). Both were significantly less stable than the W6/32-reactive class I molecules (Figure 3, lanes 24–25 and 32–33 as compared to lanes 40–41).

Thus, it appears that the Cw7 heavy chains, as compared to the A25 and B8 heavy chains, preferentially interact with calnexin both in the presence and in the absence of $\beta_2$m. Because of this and/or because of their intrinsic stability, Cw7 heavy chains preferentially accumulate in large amounts in the ER in the absence of $\beta_2$m.

We were puzzled by the stability of the Cw7-free heavy chains and wondered whether they were all bound for disposal, or, similar to HLA-Cw1 (Martayan et al, 1997) and certain murine class I molecules (Potter et al, 1984; Allen et al, 1986; Smith et al, 1993; Machold et al, 1995), at least a fraction of HLA-Cw7 molecules could be expressed on the cell surface in the absence of $\beta_2$m.

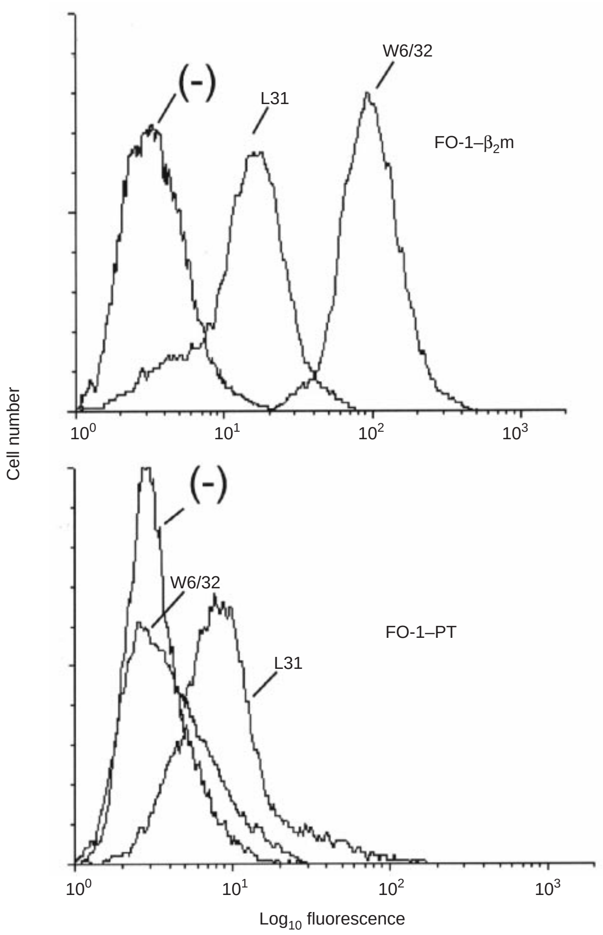

**Figure 4** Flow cytometry analysis of class I HLA molecules in FO-1 melanoma cells and their $\beta_2$m transfectants. FO-1 melanoma cells defective in $\beta_2$m (lower panel) and their $\beta_2$m transfectants (upper panel) were stained with the indicated monoclonal antibodies and with an antibody of irrelevant specificity (–) by indirect immunofluorescence. Cells were analysed in a FACScan (Becton & Dickinson) flow cytometer

### Surface expression of free heavy chains in the absence of $\beta_2$m

Both the flow cytometry results shown in Figure 4 and the surface labelling experiments shown in Figure 5 documented the presence of very low levels of L31-reacting molecules and the absence of significant levels of W6/32-reacting molecules on the surface of $\beta_2$m-defective FO-1 cells. Flow cytometry demonstrated that the levels of free heavy chains in parental FO-1 cells were approximately 40% of those in $\beta_2$m transfectants (Figure 4). In agreement with these data, metabolic labelling (Figure 5A) and cell surface iodination experiments (Figure 5B) followed by SDS-PAGE analysis demonstrated that the FO-1 parental cell line was capable of synthesizing (Figure 5, lane 2) and expressing only low molecular weight free heavy chains on its surface (Figure 5, lane 10). At variance, the FO-1–$\beta_2$m cell line synthesized and expressed two types of free heavy chains. The former, although possibly less abundant, was similar in size to the free heavy chains expressed by the parental cells. The latter type had a molecular weight typical of mature heavy chains (Figure 5, lanes 6 and 13). These results suggest that the free HLA-Cw7 heavy chains expressed on the surface of FO-1–$\beta_2$m cells include those which reach the surface in parental cells and an additional pool, presumably derived from the dissociation of heavy chain: $\beta_2$m complexes. No class I heavy chains were detected (not shown) on the cell surface of $\beta_2$m-defective cells using the HC-10 antibody (Stam et al, 1986). This confirms previous findings demonstrating (D'Urso et al, 1992) that several antibodies to $\beta_2$m-free heavy chains fail to detect class I molecules on the cell surface of FO-1 cells.

To determine whether the iodinated L31-reacting HLA-Cw7 molecules were from the cell surface or from intracellular class I molecules released or exposed by dead or damaged cells, the experiment depicted in Figure 5C was carried out. In this experiment, performed in parallel with those shown in panels A and B, the FO-1 and FO-1–$\beta_2$m cells were Dounce homogenized, and the homogenates submitted to the iodination procedure in parallel with live cells. An antibody to heat shock protein 72 (Hsp 72) was used to immunoprecipitate this cytosolic protein from metabolically labelled cells (Figure 5A, lanes 3 and 7), $^{125}$I-labelled cells

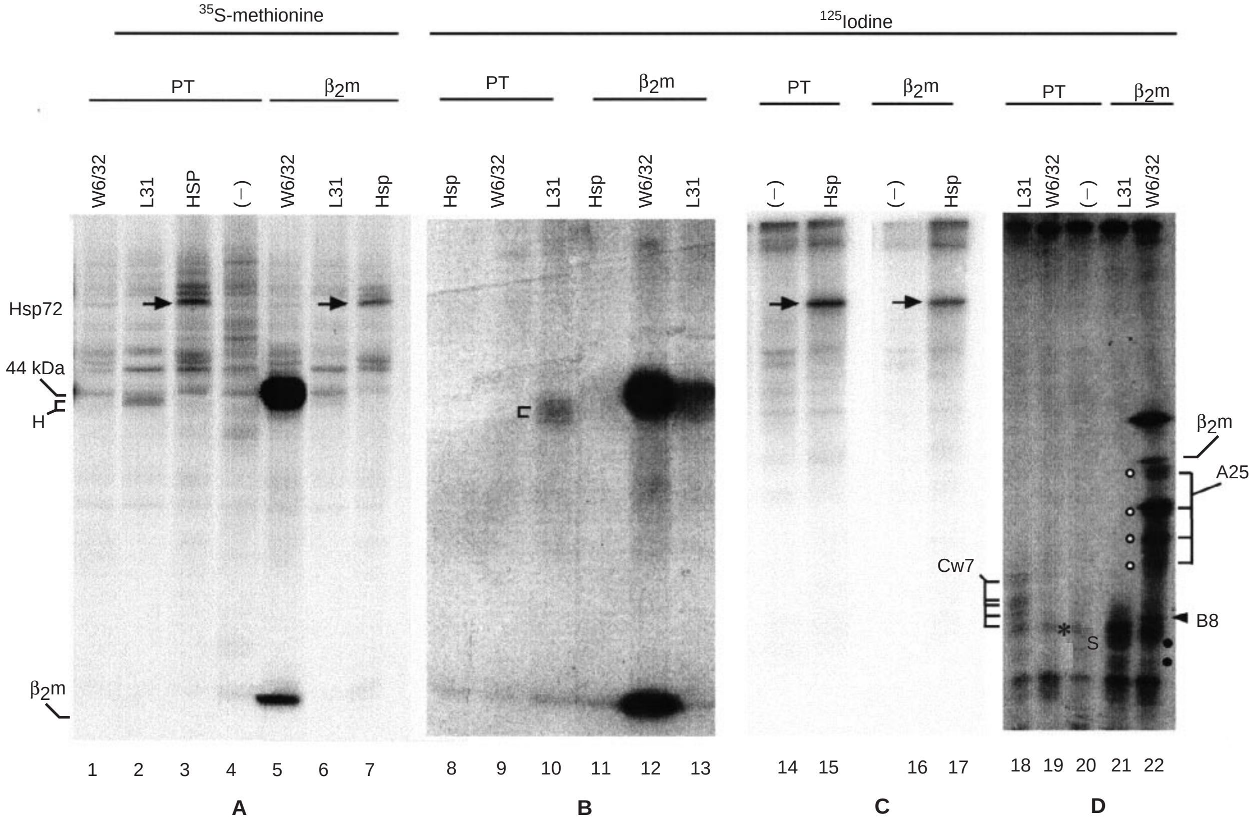

**Figure 5** Surface radio-labelling with $^{125}$I of FO-1 cells and their $\beta_2$m transfectants. Parental FO-1 cells and their $\beta_2$m transfectants, as indicated, were either metabolically labelled for 2 h with $^{35}$S-methionine (approximately 8.7 MBq ml$^{-1}$) (**A**) or extrinsically labelled with $^{125}$I (**B** and **D**), solubilized by NP40 and immunoprecipitated with the indicated antibodies to class I molecules, an antibody to the 72 kDa heat shock protein (Hsp) and an antibody of irrelevant specificity (–). Alternatively, the cells were Dounce homogenized as described in Materials and Methods, and the lysates iodinated in parallel with whole intact cells (**C**). Undigested immunoprecipitates were analysed, under reducing conditions, by either a 12.5% reducing SDS-PAGE (**A–C**) or an IEF gel (**D**). An arrow marks the Hsp72 band in lanes 3 and 7. The low molecular weight heavy chain forms are indicated (H). The basic (unsialylated) HLA-Cw7 forms in lane 18 and the sialylated bands in lane 22 are indicated as in Figure 1. Additional acidic bands are also present in lanes 21–22 (dots) which are barely visible in the IEF gels shown in Figures 1 and 3. An asterisk, as in Figure 1, marks a background band

(Figure 5B, lanes 8 and 11) and $^{125}$I-labelled Dounce homogenates (Figure 5C, lanes 15 and 17). Hsp72 was similarly visualized in metabolically labelled cells and in $^{125}$I-labelled cell homogenates (Figure 5A and C, arrows) but not in the $^{125}$I-labelled intact cells (Figure 5B). Given the large amounts of $^{125}$I-HSP72 recovered from the homogenates, it may be concluded that the lysis of even a small percentage of cells in the experiments shown in Figure 5B would be revealed by the presence of a $^{125}$I-Hsp72 band. Thus, the iodination procedure employed was specific for cell surface proteins.

In agreement with the low molecular weight of free heavy chains in $\beta_2$m-defective cells, a surface iodination experiment followed by immunoprecipitation and IEF (Figure 6D) showed that L31 identified unsialylated HLA-Cw7 heavy chains in parental FO-1 cells (Figure 6, lane 18) and mainly sialylated heavy chains in $\beta_2$m-transfected FO-1 cells (Figure 6, lane 21). Immunoprecipitation with the W6/32 antibody demonstrated the presence of sialylated HLA-A25, -B8 and Cw7 heavy chains on the surface of $\beta_2$m transfectants (Figure 6, lane 22) but not on that of parental FO-1 cells (Figure 6, lane 19). The absence of unsialylated (pre-Golgi) HLA-A25 and -B8 molecules in lane 22 (compare these patterns with those in Figure 1) further pointed to the cell surface specificity of the iodination procedure we employed. The W6/32-reactive HLA-A25 molecules were expressed on the cell surface in greater amounts than the B8 and Cw7 molecules.

### DISCUSSION

In the present study, the FO-1 human malignant melanoma cell line, previously found to be $\beta_2$m-defective (D'Urso et al, 1992), was HLA-genotyped and further characterized at a biochemical level. Surprisingly, transfection with $\beta_2$m did not result in the complete correction of the defect in class I expression of FO-1 cells, suggesting the presence of additional defects in class I expression/antigen processing.

One such defect was identified as the selective down-regulation of the class I-dedicated TAP chaperone (Solheim et al, 1997*a*), as opposed to the normal levels of the promiscuous chaperones, calnexin and calreticulin. Due, at least in part, to the extremely low TAP levels, the class I:TAP interaction was not efficient. Consequently, heavy chain:$\beta_2$m dimers were formed, but were thermally unstable, a feature considered to be the hallmark of defective peptide loading and incomplete assembly of class I molecules (Elliott, 1997).

At variance from TAP, the steady-state levels of calreticulin were normal in FO-1 cells, and $\beta_2$m was present in excess amounts

 © Cancer Research Campaign 1999

in FO-1–$\beta_2$m transfectants. However, neither class I molecules nor $\beta_2$m were capable of efficient interactions with calreticulin. This appears to be qualitatively different from most of the available examples (Seliger et al, 1997) of defects in class I expression of neoplastic cells, known to reflect a true down-regulation of members of the antigen-processing machinery.

It might be argued that a limiting stoichiometry of the TAP complex may adversely affect the class I:calreticulin interaction, thereby providing a common denominator for the two defects. However, this seems unlikely because the class I:$\beta_2$m dimer binds calreticulin before binding to the TAP complex in the currently accepted model of class I assembly (Elliott, 1997). Moreover, class I:calreticulin complexes have been shown to form normally in a TAP-defective cell line (Sadasivan et al, 1996). Thus, the defects impairing the interactions of class I heavy chains with TAP and calreticulin are most likely distinct. At the present time, we do not know if the poor association of class I molecules with calreticulin is due to a defect in a component of the antigen-processing machinery distal to calnexin. It is intriguing to speculate that FO-1 cells might lack tapasin, a recently described molecule which participates in the assembly of class I molecules (Ortmann et al, 1997). A down-regulation of tapasin might increase the turnover of TAP molecules, thus explaining the low steady-state levels of TAP found in FO-1 cells. Further experiments are required to address this issue.

Another putative defect of FO-1 cells may be the loss of one HLA-A, -B, -C haplotype. Because no more than three classical class I alleles could be PCR-amplified from genomic DNA, either FO-1 cells were derived from a HLA-homozygous donor or they lost one haplotype, retaining only a hemizygous set of HLA-A, -B, -C molecules. Due to the high number of class I alleles present at the population level, the latter possibility is more likely. Only further investigations will clarify this issue.

Thus, excluding the putative haplotype loss, a minimum of three defects hinders class I expression in FO-1 cells: the lack of $\beta_2$m, a low expression of TAP and a poor class I:calreticulin interaction. This variety of defects further adds to the complexity of the events which may impair antigen presentation in a given tumour cell line. Specifically, our results show that different alleles are affected in different ways by the same defect(s) in class I assembly. For instance, the A25 allele is surface-expressed at higher levels than the B8 and Cw7 alleles in FO-1–$\beta_2$m cells. Although the number of W6/32-reacting molecules on the cell surface may not necessarily reflect the number of peptide-filled (functional) class I molecules, our results suggest that allele- and locus-specific features of the class I heavy chains may determine which class I molecules withstand damage to the antigen-processing machinery. This may ultimately affect the repertoire of the residual CTL and NK responses against class I-defective tumour cells.

A detailed characterization of the defects affecting class I processing in FO-1 cells was not attempted in the present study. Nevertheless, FO-1 cells exemplify a deranged biosynthesis of class I molecules. The lack or drastic reduction of some biologically relevant protein:protein interactions provides a clear background against which the preferential interaction of calnexin with HLA-Cw7 can be viewed. It has been recently shown that certain HLA-C alleles display a more selective peptide binding and a stronger interaction with the TAP complex than certain HLA-A and -B alleles. Retention in the ER by the TAP complex may then account for the low surface expression of HLA-C:$\beta_2$m complexes (Neisig et al, 1998). We found that at least two additional forms of HLA-C molecules are refractory to degradation in FO-1 cells and preferentially accumulate in the ER: the former $\beta_2$m-free but complexed with calnexin; the latter free of both $\beta_2$m and calnexin. At least this latter form is consistently present in a variety of cells and in normal and neoplastic tissues (Giacomini et al, 1997). A coordinated expansion of three intercommunicating molecular pools of heavy chains (calnexin-bound, calnexin-free and TAP-bound heavy chains) in cells capable of supporting optimal class I:chaperone interactions may have been selected during evolution to retard the transport of HLA-C. Perhaps the long permanence and accumulation of HLA-C in the ER may be considered an adaptive mechanism to compensate for its selectivity in peptide binding. In addition, or alternatively, the various HLA-C assembly intermediates may represent a reservoir of class I molecules awaiting peptide loading. If so, alternative ligands might be rescued from cellular proteins which, for some reason, failed to serve as ligand donors for HLA-A and -B class I molecules.

The complete loss of class I expression is quite rare in human neoplasm, which frequently undergo less severe losses (Garrido et al, 1993). There is evidence that $\beta_2$m and/or antigenic peptides are available in limiting amounts in a significant percentage of melanoma cells (Wang et al, 1996). The considerable stability of HLA-C polypeptides in $\beta_2$m-defective FO-1 cells suggests that HLA-C may be less affected than HLA-A and -B heavy chains by imbalances in the three subunits of the class I HLA complex. Thus, HLA-C, although selective in its ability to bind peptides and expressed at low levels on the cell surface, may be an important recognition element for immune effectors (both CTLs and NK cells) in tumours which retain suboptimal class I expression levels.

Some biochemical features of HLA-Cw7 molecules in $\beta_2$m-defective cells (its lack of reactivity with antibodies to conformed heavy chains and expression on the cell surface) are reminiscent of murine H2–$D^b$ and –$L^d$ molecules (Potter et al, 1984; Allen et al, 1986). This indicates that surface expression in the absence of $\beta_2$m is the prerogative of a group of class I alleles which is largely independent of the animal species, cell lineage and culture conditions. No free HLA-A, -B, -C heavy chains have been detected at the surface of most $\beta_2$m-defective cell lines described to date (Fellous et al, 1977; D'Urso et al, 1992; Gattoni-Celli et al, 1992; Wang et al, 1993; Bicknell et al, 1994; Porgador et al, 1997). In a recent report (Wang et al, 1998) the surface expression of HC-10-reacting, $\beta_2$m-free HLA-A25 and -B8 heavy chains was detected in FO-1 cells. This, however, required treatment with IFN-$\alpha$ and hypothermia. Our results complement the available information, since they indicate that the constitutive surface expression of free heavy chains in the absence of $\beta_2$m is a distinctive feature of HLA-C molecules which may be observed only in certain cell lines. Accordingly, we recently observed similar low surface levels of free HLA-Cw1 heavy chains in the $\beta_2$m-defective KJ29 kidney carcinoma cell line (Martayan et al, 1997), and of free HLA-Cw3 and -Cw6 heavy chains in the Burkitt's lymphoma Daudi cell line (our own unpublished results). Our data and the published literature on the subject suggest that the massive intracellular accumulation of HLA-C molecules might have an important role in facilitating this event. The surface expression of free heavy chains may then be considered a result of the 'escape' from the ER of a few unfolded molecules, i.e. an epiphenomenon of intracellular accumulation whose importance must still be established.

We have been able to demonstrate that, differently from murine heavy chains, essentially all the free HLA-Cw7 heavy chains expressed on the surface of parental FO-1 cells are unsialylated and display a low molecular weight. These features, taken together, are clearly indicative of an 'immature' oligosaccharide processing and unorthodox intracellular transport. Thus, a limited but significant transport to the cell surface occurs in the case of free HLA-Cw7 heavy chains even when they do not complete two basic maturation steps, i.e. assembly and terminal glycosylation. Further studies are warranted to establish whether the glycosylation intermediates of HLA-Cw7 detected in this study have a distinctive ability to negotiate the exocytic path and may also trigger CTL and NK responses. In this regard, it should be recalled that certain NK receptors have a carbohydrate recognition domain (Long and Wagtmann, 1997).

### ACKNOWLEDGEMENTS

Supported by the Program for Finalized Research (1998) of the Italian Ministry of Health (PG and GBF), CNR Target Project on Biotechnology 1998 (funds for two units), and a grant from Istituto Superiore di Sanità 1996 (Sostituzioni funzionali, organi artificiali e trapianti di organo). The authors are grateful to Drs A Siccardi and A Beretta, Dibit S Raffaele, Milano, for the generous gift of antibody L31; to Dr Hidde Ploegh, MIT, Cambridge, USA for HC-10; to Dr J Trowsdale, Imperial Cancer Research Fund, London, UK, for the polyclonal antibodies to TAP1 and TAP2; to Drs M Fiscella and E Appella, NCI, NIH, for the pCMV–$\beta_2$m clone; to Dr Marco Colonna, Basel Institute for Immunology, for the 221.Cw1 transfectant, and to Dr Paul B Fisher, Columbia University, New York, for affinity purified, recombinant IFN-γ. We are grateful to Gillian McIntire for her assistance with the English text and to Maria Vincenza Sarcone for her secretarial assistance.

### REFERENCES

Allen H, Fraser J, Flyer D, Calvin S and Flavell R (1986) $\beta_2$-microglobulin is not required for cell surface expression of the murine class I histocompatibility antigen H-2D$^b$ or of a truncated H-2D$^b$. *Proc Natl Acad Sci USA* **83**: 7447–7451

Bicknell DC, Rowan A and Bodmer WF (1994) $\beta_2$-microglobulin mutations: a study of established colorectal cell lines and fresh tumors. *Proc Natl Acad Sci USA* **91**: 4751–4755

Cook RJ, Solheim JC, Connolly JM and Hansen TH (1995) Induction of peptide-specific CD8$^+$ CTL clones in $\beta_2$-microglobulin deficient mice. *J Immunol* **154**: 47–57

D'Urso CM, Wang Z, Cao Y, Tatake R, Zeff RA and Ferrone S (1992) Lack of HLA class I antigen expression by cultured melanoma cell FO-1 due to a defect in $\beta_2$m gene expression. *J Clin Invest* **87**: 284–292

Degen E and Williams D (1991) Participation of a novel 88-kD protein in the biogenesis of murine class I histocompatibility molecules. *J Cell Biol* **112**: 1099–1115

Elliott T (1991) How do peptides associate with MHC class I molecules? *Immunol Today* **12**: 386–388

Elliott T (1997) How does TAP associate with MHC class I molecules? *Immunol Today* **18**: 375–379

Fellous M, Kamoun M, Wiels J, Dausset J, Clements G, Zeuthen J and Klein G (1977) Induction of HLA expression in Daudi cells after cell fusion. *Immunogenetics* **5**: 423–436

Garrido F, Cabrera T, Concha A, Glew S, Ruiz-Cabello F and Stern PL (1993) Natural history of HLA expression during tumour development. *Immunol Today* **14**: 491–499

Gattoni-Celli S, Kirsch K, Timpane R and Isselbacher KJ (1992) $\beta_2$-microglobulin gene is mutated in a human colon cancer cell line (HCT) deficient in the expression of HLA class I antigens on the cell surface. *Cancer Res* **52**: 1201–1204

Giacomini P, Beretta A, Nicotra MR, Ciccarelli G, Martayan A, Cerboni C, Lopalco L, Bini D, Delfino L, Ferrara GB, Siccardi AG and Natali PG (1997) HLA-C heavy chains free of $\beta_2$m: distribution in normal tissues and neoplastic lesions of non-lymphoid origin and IFN-γ responsiveness. *Tissue Antigens* **50**: 555–566

Hajek-Rosenmayr A, Jungl L, Stammler M and Kirnbauer M (1989) HLA-C locus antigens seen by one-dimensional isoelectric focusing: definition of the so far known HLA-C specificities and of two subtypes. *Hum Immunol* **26**: 227–236

Hansen TH and Lee DR (1997) Mechanism of class I assembly with $\beta_2$ microglobulin and loading with peptide. *Adv Immunol* **64**: 105–137

Hansen TH, Myers NB and Lee DR (1988) Studies of two antigenic forms of L$^d$ with disparate $\beta_2$-microglobulin associations suggest that $\beta_2$m facilitates the folding of the α1 and α2 domains during de novo synthesis. *J Immunol* **140**: 3522–3527

Lehmann-Grube F, Dralle H, Utermohlen O and Lohler J (1994) MHC class I molecule-restricted presentation of viral antigen in $\beta_2$-microglobulin-deficient mice. *J Immunol* **153**: 595–603

Long EO and Wagtmann N (1997) Natural killer cell receptors. *Curr Opin Immunol* **9**: 344–350

Machold RP, Andrée S, Van Kaer L, Ljunggren H-G and Ploegh HL. (1995) Peptide influences the folding and intracellular transport of free major histocompatibility complex class I heavy chains. *J Exp Med* **181**: 1111–1122

Markwell MA and Fox CF (1978) Surface specific iodination of membrane proteins of viruses and eukaryotic cells using 1,2,3,4 tetrachloro-3α, 6β-diphenylglycoluril. *Biochemistry* **17**: 4807–4817

Martayan A, Fiscella M, Setini A, Ciccarelli G, Gambari R, Feriotto G, Beretta A, Siccardi AG, Appella E and Giacomini P (1997) Conformation and surface expression of free HLA-CW1 heavy chains in the absence of $\beta_2$-microglobulin. *Hum Immunol* **53**: 23–33

Neefjes JJ and Ploegh HL (1988) Allele and locus-specific differences in cell surface expression and the association of HLA class I heavy chain with $\beta_2$-microglobulin: differential effects of inhibition of glycosylation on class I subunit association. *Eur J Immunol* **18**: 801–810

Neefjes JJ, Breur-Vriesendorp BS, Seventer I, Ivanyi P and Ploegh HL (1986) Improved biochemical method for the analysis of HLA-class I antigens. Definition of new HLA-class I subtypes. *Immunology* **16**: 169–181

Neisig A, Melief CJM and Neefjes JJ (1998) Reduced cell surface expression of HLA-C molecules correlates with restricted peptide binding and stable TAP interaction. *J Immunol* **160**: 171–179

Ortmann B, Copeman J, Lehner PJ, Sadasivan B, Herberg JA, Grandea AG, Riddell SR, Tampé R, Spies T, Trowsdale J and Cresswell P (1997) A critical role for tapasin in the assembly and function of multimeric MHC class I-TAP complexes. *Science* **277**: 1306–1309

Parham P, Barnstable CJ and Bodmer WF (1979) Use of monoclonal antibody (W6/32) in structural studies of HLA-A,B,C antigens. *J Immunol* **23**: 342–349

Pellegrino MA, Ng AK, Russo C and Ferrone S (1982) Heterogenous distribution of determinants defined by monoclonal antibodies on HLA-A, -B antigen bearing molecules. *Transplantation* **34**: 18–23

Pera C, Delfino L, Morabito A, Longo A, Johnston-Dow L, White CB, Colonna M and Ferrara GB (1997) HLA-A typing: comparison between serology, the amplification refractory mutation system with polymerase chain reaction and sequencing. *Tissue Antigens* **50**: 372–379

Porgador A, Mandelboim O, Restifo NP and Strominger JL (1997) Natural killer cell lines kill autologous $\beta_2$m-deficient melanoma cells: implications for cancer immunotherapy. *Proc Natl Acad Sci*, **94**: 13140–13145

Potter TA, Boyer C, Schmitt-Verhulst AM, Goldestein P and Rajan TV (1984) Expression of H-2D on the cell surface in the absence of detectable $\beta_2$-microglobulin. *J Exp Med* **84**: 317–325.

Prasad VK and Yang SY (1996) Allele assignment for HLA-A, -B, and -C genes to the Tenth International Histocompatibility Workshop cell lines. *Tissue Antigens* **47**: 538–546

Rajagopalan S and Brenner MB (1994) Calnexin retains unassembled major histocompatibility complex class I free heavy chains in the endoplasmic reticulum. *J. Exp. Med.* **180**: 407–412

Sadasivan B, Lehner PJ, Ortmann B, Spies T and Cresswell P (1996) Roles for calreticulin and a novel glycoprotein, tapasin, in the interaction of MHC class I molecules with TAP. *Immunity* **5**: 103–114

Salter RD and Cresswell P (1986) Impaired assembly and transport of HLA-A and -B antigens in a mutant TxB cell hybrid. *EMBO J* **5**: 943–949

Seliger B, Maeurer MJ and Ferrone S (1997) TAP off – tumors on. *Immunol Today* **18**: 292–299

Setini A, Beretta A, De Santis C, Meneveri R, Martayan A, Mazzilli MC, Appella E, Siccardi AG, Natali PG and Giacomini P (1996) Distinctive features of the α1 domain α helix of HLA-C heavy chains free of β2-microglobulin. *Hum Immunol*, **46**: 69–81

 © Cancer Research Campaign 1999

Shimizu Y and Demars R (1989) Production of human cells expressing individual transferred HLA-A,-B,-C genes using an HLA-A, -B,-C null human cell line. *J Immunol*, **142**: 3320–3328

Smith JD, Myers NB, Gorka J and Hansen TH (1993) Model for the in vivo assembly of nascent $L^d$ class I molecules and for the expression of unfolded $L^d$ molecules at the cell surface. *J Exp Med*, **178**: 2035–2046

Solheim JC, Carreno BM and Hansen TH (1997*a*) Are transporter associated with antigen processing (TAP) and Tapasin class I MHC chaperones? *J Immunol* **158**: 541–543

Solheim JC, Harris MR, Kindle CS and Hansen TH (1997*b*) Prominence of $\beta_2$-microglobulin, class I heavy chain conformation, and tapasin in the interactions of class I heavy chain with calreticulin and the transporter associated with antigen processing. *J Immunol*, **158**: 2236–2241

Stam NJ, Spits H and Plogh HL (1986) Monoclonal antibodies raised against denatured HLA-B locus heavy chains permit biochemical characterization of certain HLA-C products. *J Immunol*, **137**: 2299–2306

Wang Z, Cao Y, Albino P, Zeff RA, Houghton AN and Ferrone S (1993) Lack of HLA class I antigen expression by melanoma cell SK-MEL-33 caused by a reading frameshift in $\beta_2$-microglobulin messenger RNA. *J Clin Invest* **91**: 684–692

Wang Z, Margulies D, Hicklin J and Ferrone S (1996) Molecular and functional phenotypes of melanoma cells with abnormalities in HLA class I antigen expression. *Tissue Antigens* **47**: 382–390

Wang ZG, Arienti F, Parmiani G and Ferrone S (1998) Induction and functional characterization of β2-microglobulin ($\beta_2$-μ)-free HLA class I heavy chains expressed by $\beta_2$-μ-deficient human FO-1 melanoma cells. *Eur J Immunol* **28**: 2817–2826

Yang SY (1989) A standardized method for detection of HLA-A and HLA-B alleles by one-dimensional isoelctricfocusing (IEF) gel electrophoresis. In *Immunobiology of HLA*, Dupont B (ed). pp. 332–335. Springer-Verlag: New York

© Cancer Research Campaign 1999
